# Supplementary material for: Gate-tunable negative longitudinal magnetoresistance in the predicted type-II Weyl semimetal WTe2
Source: Nat Commun. 2016 Oct 11;7:13142. doi: 10.1038/ncomms13142 (PMC5062597; doi:10.1038/ncomms13142)
Supplement: Supplementary Information — Supplementary Figures 1-9 and Supplementary Table 1 [file ncomms13142-s1.pdf]

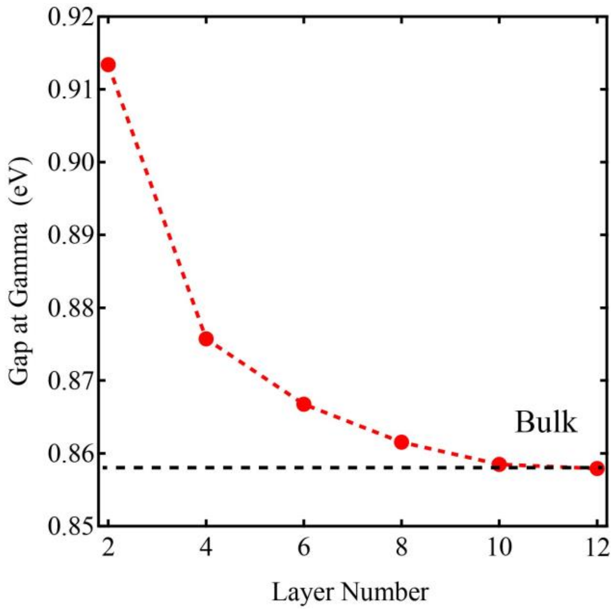

Supplementary Figure 1. Layer number dependence of the band gap at Γ (0, 0, 0) point of WTe_2. The energy bands of layered materials are generally sensitive to the thickness (layer number) when approaching 2D. Because Weyl fermions cannot exist in a 2D system, to explore the lower band thickness appropriate for our studies, the gap at Γ (0, 0, 0) of the Brillouin Zone of WTe_2 with different layer numbers were calculated. A vacuum spacing of 15 Å was used so that the interaction in the non-periodic directions could be neglected. The band gap at Γ point is sensitive to the layer number and tends to be constant when the layer number approaches 10 (approximately 7 nm), suggesting that the films thicker than 7 nm share energy bands similar to those of the bulk samples, permitting the existence of Weyl points. This result is consistent with our experimental observation that the negative longitudinal MR effect is only observed in the 7-15-nm-thick samples and not in samples thinner than 7 nm.

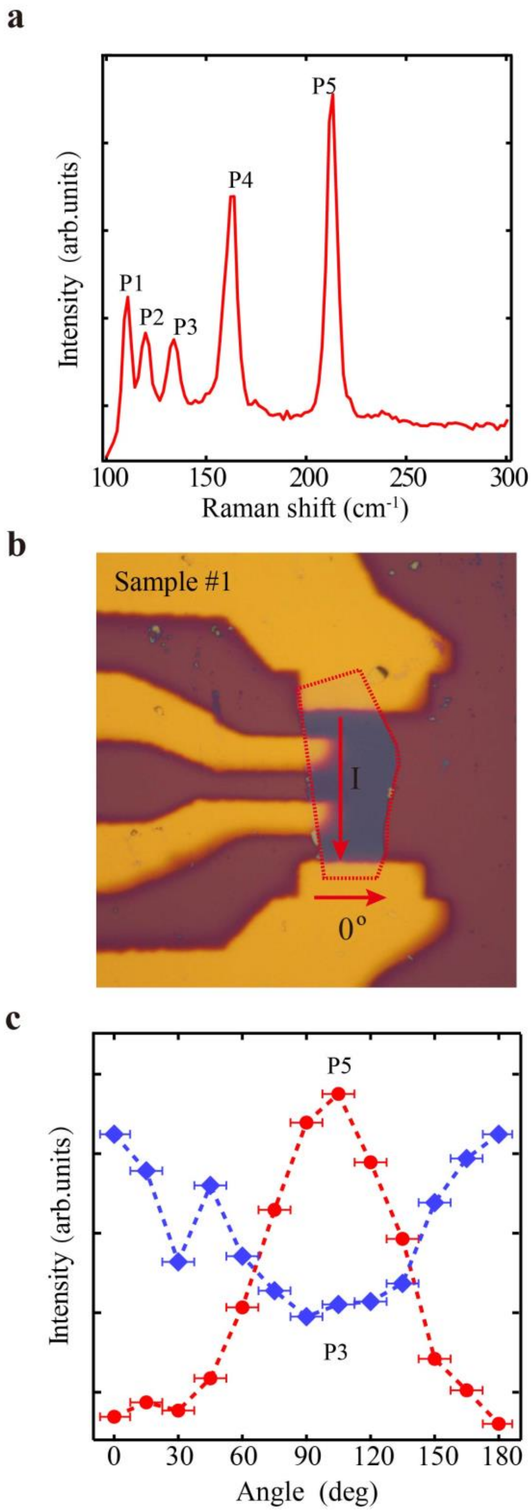

Supplementary Figure 2. Polarized Raman scattering spectra of sample #1. (**a**) Non-polarized Raman scattering spectra. The measurements were performed using a 514-nm excitation laser at room temperature. (**b**) Device picture with angle definition as marked by the red arrows. The dashed red lines indicate the shape of the flake. (**c**)

Angle dependence of the intensities of P3 and P5 of sample in (**b**), which is measured with the incident polarization vector (***e*i**) paralleling to the scattered polarization vector (***e*s**). The error bar is approximately ±7.5° which comes from the accuracy of measurement. Since the intensities of P3 and P5 are maximized and minimized, respectively, when ***e*i** and ***e*s** are parallel to the ***a*** axis. The direction of 0° in (**b**) can be determined to be along the ***a*** axis and the current direction is along the ***b*** axis.

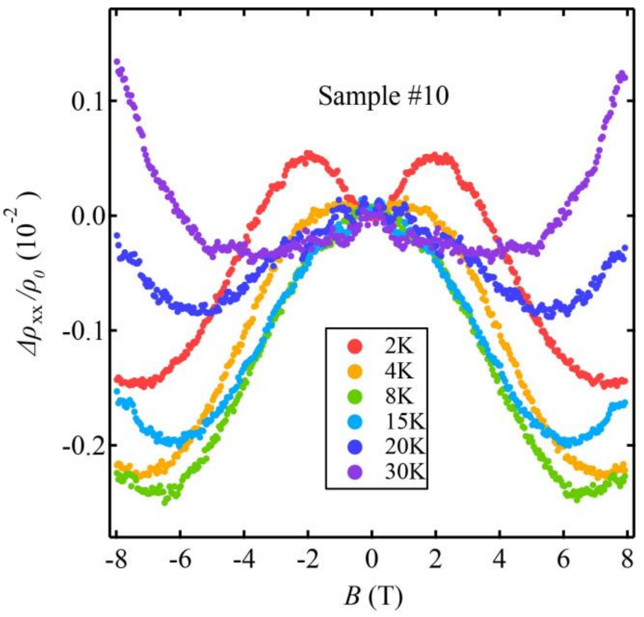

Supplementary Figure 3. Negative longitudinal MR of sample #10 measured at different temperatures with B//I//b. The WAL effect almost disappears at 8 K, while the negative longitudinal MR persists at much higher temperature (up to 30 K), indicating that the negative longitudinal MR is less temperature-sensitive than the WAL effect.

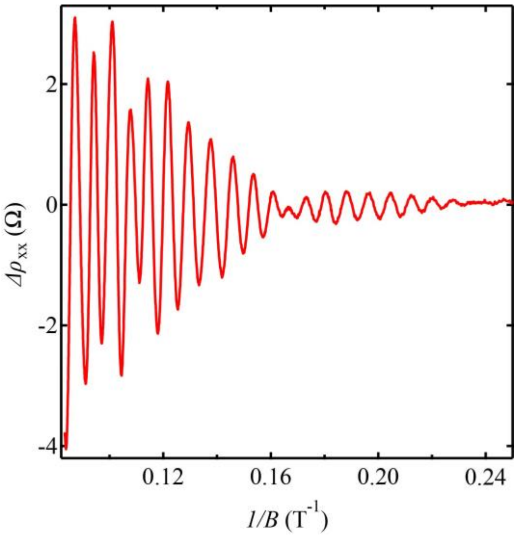

Supplementary Figure 4. SDH oscillation data of sample #4.

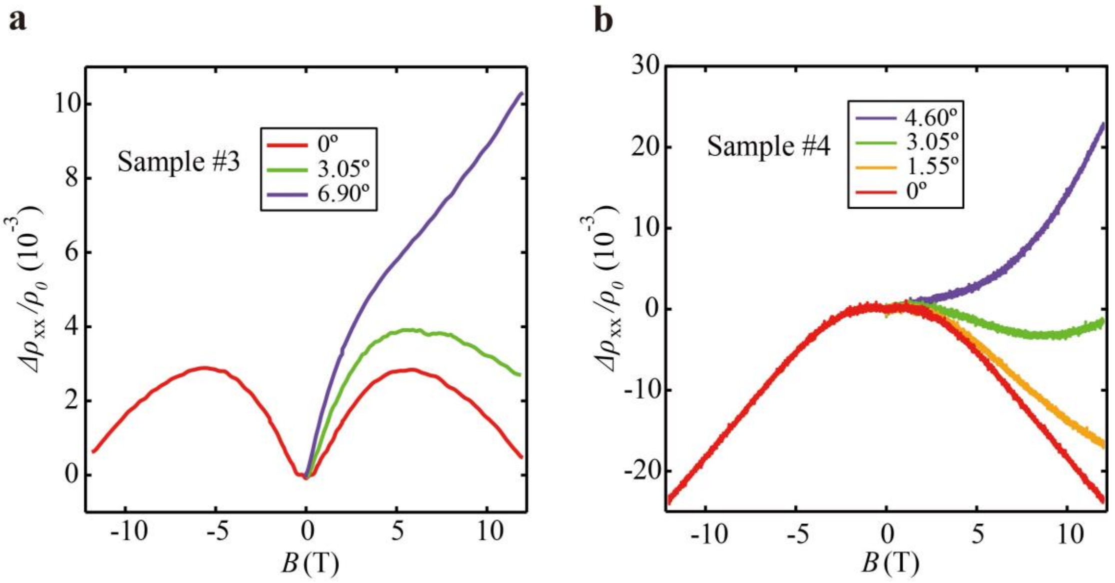

Supplementary Figure 5. Angle-dependent negative longitudinal MR of additional samples. (a) and (**b)** show the datasets of samples #3 and #4, respectively. Both samples were measured with current along the b axis and exhibit strong angle θ sensitivity.

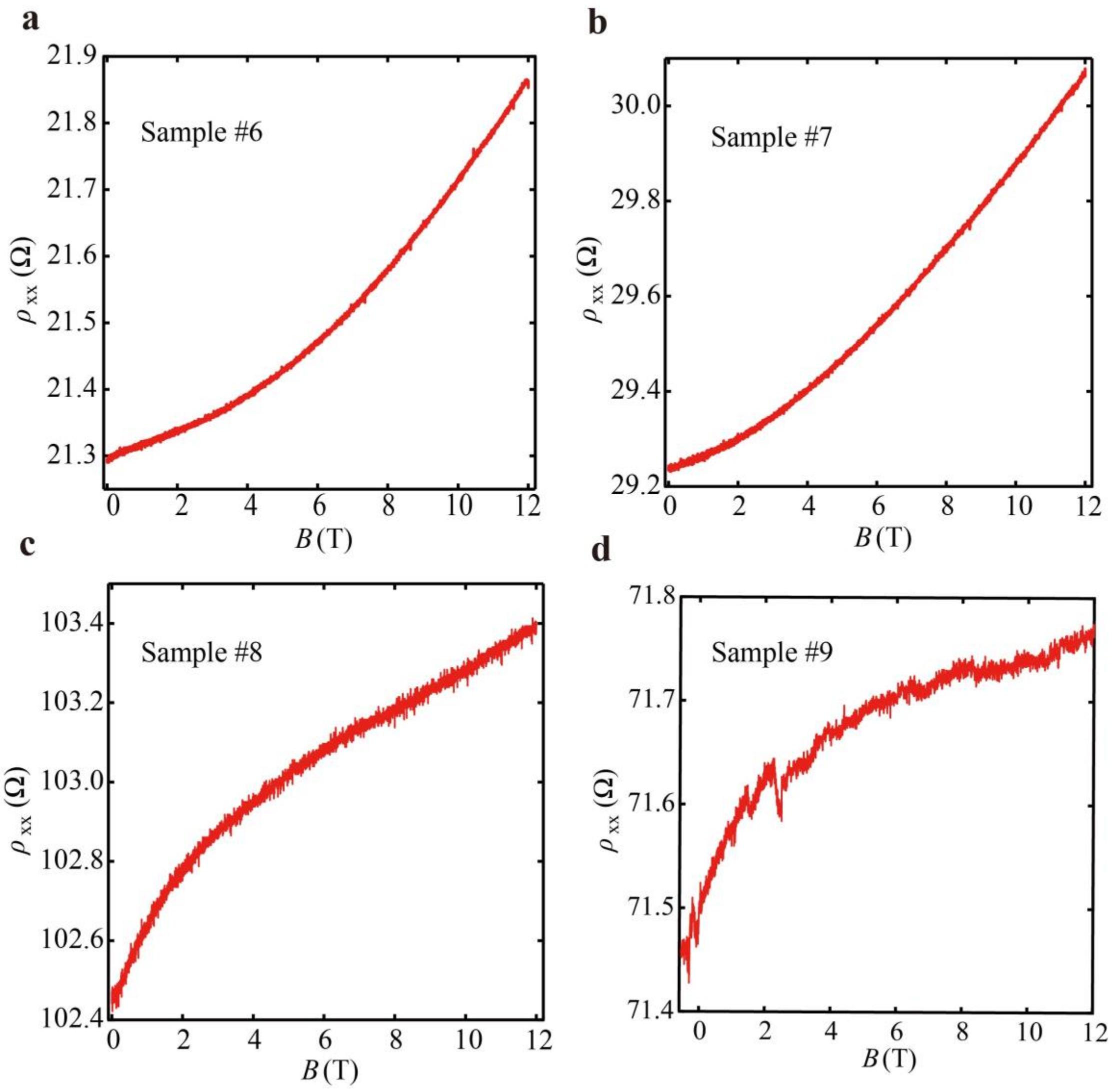

Supplementary Figure 6. Longitudinal MR of all measured samples with current along the *a* axis. The samples were prepared under very similar conditions (exfoliated from the same batch of single crystals and within the thickness range of 7-15 nm). Only positive longitudinal MR is observed in all 4 samples, supporting the predicted planar orientation dependence of the type-II Weyl fermion chiral anomaly in thin WTe_2 films.

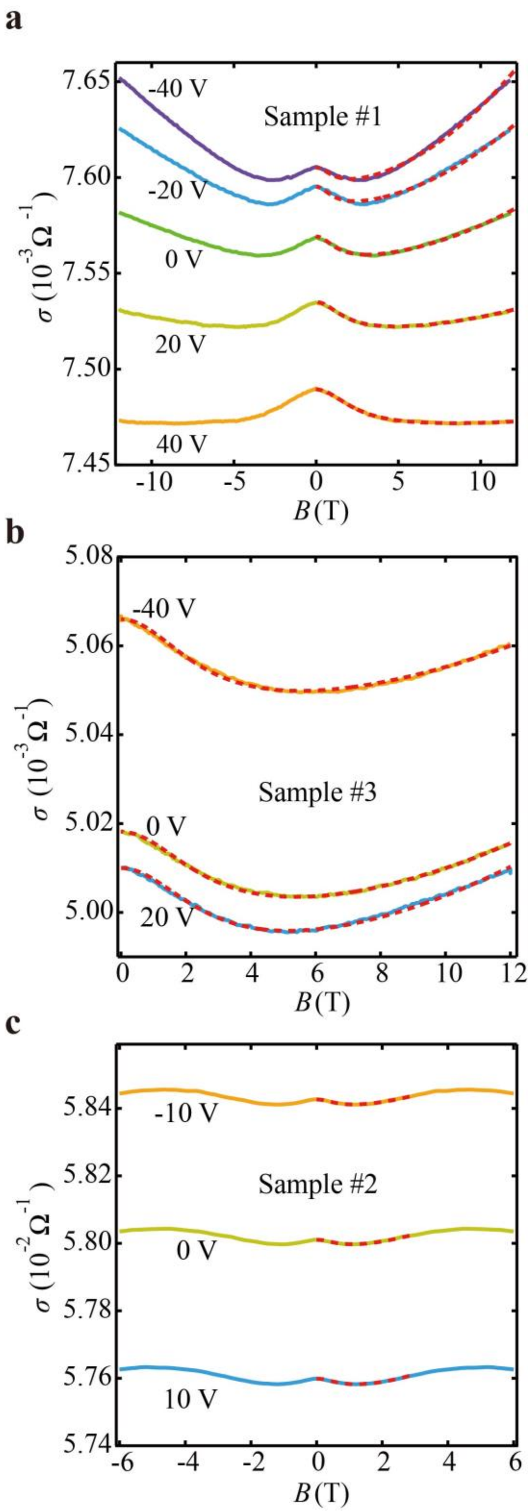

Supplementary Figure 7. Fitting results of different samples at different V_{bg}. The solid lines and red dashed lines are the experimental and fitting curves, respectively. For sample #2, because of the contribution of positive longitudinal MR at high magnetic fields, we only fitted the negative longitudinal region (0-3 T).

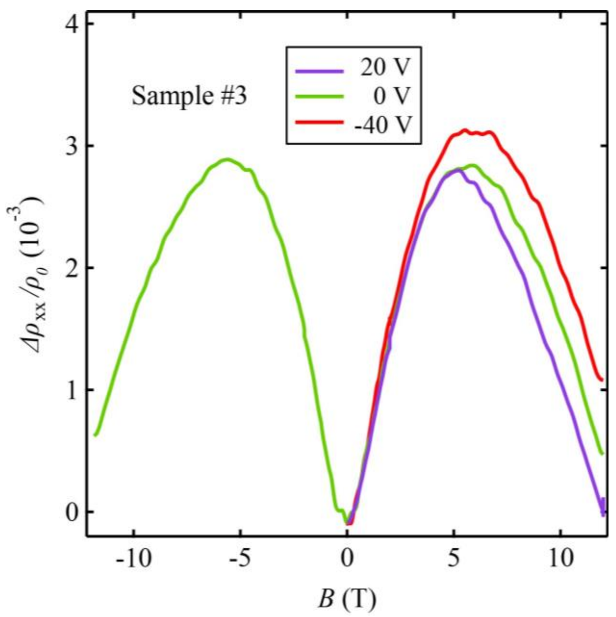

Supplementary Figure 8. Negative longitudinal MR of sample #3 for various V_{bg}.

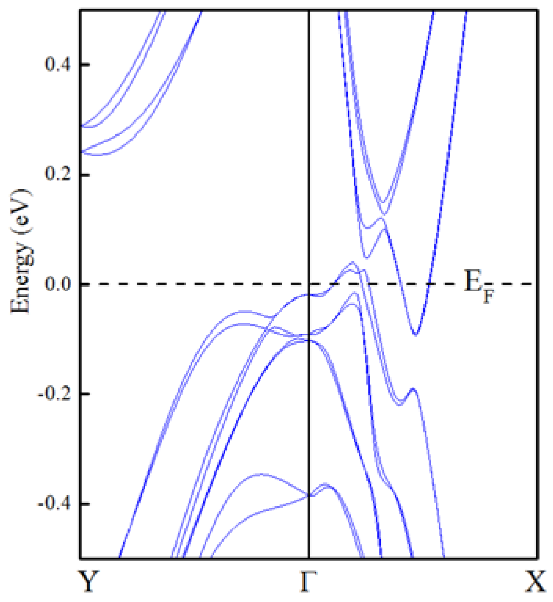

Supplementary Figure 9. Band structure of WTe_2 along the Y-Γ-X direction with SOC.

Sample	Landau level Index N	F (T)
sample #1	10	112.92
sample #2	11	122.07
sample #4	14	149.54

Supplementary Table 1. Landau level index of three samples exhibiting negative longitudinal MR. The Landau level index of various samples were calculated by analyzing the SDH oscillation data. The Landau level index can be obtained from the Onsager relation $S_{\mathrm{F}}(B) = \frac{2\pi eB}{\hbar}(N + \gamma)$, where $S_{\mathrm{F}} = 2\pi eF/\hbar$, and F is the frequency of the SDH oscillations. Because of the complete Fermi surface and sample-dependent Fermi energy, the obtained frequencies are different for various samples. The Landau levels of three samples were calculated with an applied magnetic field of approximately 11 T.
